# The Effect of Simulation Training on Enhancing Nursing Students' Perceptions to Incorporate Patients' Families Into Treatment Plans: A Randomized Experimental Study

**DOI:** 10.7759/cureus.44152

**Published:** 2023-08-26

**Authors:** Amal I Khalil, Neama Y Hantira, Hend A Alnajjar

**Affiliations:** 1 Psychiatry and Mental Health Nursing, College of Nursing, King Saud Bin Abdulaziz University for Health Sciences, Jeddah, SAU; 2 Faculty of Nursing, Menoufia University, Shibin el Kom, EGY; 3 College of Nursing, King Saud Bin Abdulaziz University for Health Sciences, Jeddah, SAU; 4 Faculty of Nursing, Alexandria University, Alexandria, EGY

**Keywords:** simulation training, nursing students, rubric, training, family, patients, simulation

## Abstract

Introduction: As clinical placement in bachelor’s nursing programs becomes increasingly difficult, simulation is becoming increasingly common to enhance learning. Blended learning incorporating simulation videos provides students with the opportunity to observe and learn from exemplary practices while bridging the gap between theoretical knowledge and its practical application. This study aimed to investigate the effect of simulation training on enhancing nursing students' perception of integrating patient's families' assessments into their treatment plan.

Methods: A quantitative, experimental research design was used, with a control (56) and intervention group (67) from levels 7 and 8 senior nursing students at King Saud Bin Abdulaziz University for Health Sciences, College of Nursing, Jeddah, assigned randomly to each group. The tool consists of three sections: personal information, a Van Gelderen family rubric, and a role-play survey. The validity and reliability of the tools were confirmed by the original developer. In the current study, the reported Cronbach’s alpha was 95%.

Results: A total of 123 students participated in the study. Their ages ranged between 19 and 23 years and 23 years and above, with a mean age of 21.3 ± 1.3 among the control group and 22.2 ± 1.1 among the experimental group. There was an improvement in the mean scores in the post-training phase compared to the pre-training phase in the experimental group, with a statistically significant difference at p < 0.05. However, there were no significant differences noted between the control and experimental groups in the pre-training phase compared to the statistically significant difference noted between the two groups in the post-training phase.

Conclusion and recommendations: The findings of the study indicated that the utilization of scenario-based standardized patient-simulated exercises, guided by dedicated faculty and accompanied by reflective debriefing exercises, proved to be an effective approach for bridging the gap between theoretical knowledge and its application in clinical practice. Therefore, the study prompts curriculum revisions to incorporate family assessment into nursing practices, as well as evidence-based strategies, such as learning activities that use standardized patient or high-fidelity simulation technology to address and possibly reduce the theory-practice gap for graduates when entering clinical practice.

## Introduction

Undergraduate nurses’ clinical preparation has a direct effect on patient outcomes [1]. Therefore, nursing educators and schools must ensure that nurses have the necessary skills before entering the profession [2]. Nursing schools are responsible for preparing students to become safe and competent practicing nurses by providing them with opportunities to have diverse clinical experiences. Undergraduate nursing programs have been criticized repeatedly for the missing family curricular thread through their undergraduate baccalaureate nursing programs [3]. Moreover, there are various challenges facing nursing practice, promoting family health and social justice within the practice environment, bridging this gap, and enhancing family nursing practice.

Additionally, there is a shortage of clinical sites necessary for student learning experiences and increased patient acuteness in many inpatient mental health units, which causes an increase in nursing students’ anxiety levels. As a result, the purpose of this study was to determine whether simulation training can be an effective way for undergraduate nursing students to become more aware of patients' families and integrate them into their treatment plans. The simulation method is defined as “a pedagogy that focuses on one or more typologies to promote, improve, or validate learning from novice to expert” while the standardized patient (SP) term is defined as "an individual trained to consistently portray a patient or other individual in a scripted scenario" and "high fidelity" is defined as "experiences with standardized patients that are extremely realistic and provide a high level of interaction and realism for the learner" [4].

The main principle of using simulation in nursing education is to replicate crucial features of a clinical situation so that students can learn in a non-threatening environment [5]. Many nursing schools use simulations as an educational tool. However, there is a gap in the literature regarding whether stimulation can be used to teach family assessment and communication skills to undergraduate nursing students [6]. Not all nursing curricula currently teach the nursing interventions necessary to provide families with client care. Research has shown that family nursing care is vital in support of the patient and family unit within healthcare practices [7-9]. Over the last 20 years, family nursing care has developed as a way of thinking about and working with families [10].

Families have traditionally received limited attention in nursing curricula and healthcare institutions, with a predominant focus on enhancing patient care [11]. However, healthcare leaders are increasingly recognizing the potential of family-centered care to yield improved health outcomes and reduced costs compared to conventional hospital-centered approaches [11]. This shift is driven by various factors such as changing healthcare policies, economic considerations, technological advancements, shorter hospital stays, and healthcare movement from hospitals to community and family settings [12].

Research by Friedman et al. [13] highlights the significant influence that families, as social institutions, exert on an individual's health. Families play a critical role in providing support to patients and acting as their voice when they are unable to communicate [14]. It is crucial for families to receive information, reassurance, and proximity to their loved ones during the healthcare process [15]. However, nurses often underestimate their role in addressing the needs of family members [16].

Simulation has been widely recognized in the nursing education literature as a valuable tool that positively enhances educational outcomes [14]. However, there is a scarcity of research resources specifically focusing on the utilization of simulation to develop family nursing skills. This gap highlights the need for the development of a family assessment and communication rubric specifically tailored for simulation-based learning. Another crucial aspect to consider is the significance of debriefing tools and providing feedback to students following their participation in simulated scenarios. Despite the valuable contribution of simulation, nursing clinical education heavily relies on interactions with actual patients [14]. Incorporating debriefing and feedback mechanisms into simulation experiences is essential for optimizing learning outcomes and bridging the gap between simulation and real-world patient encounters. By addressing these aspects and utilizing simulation effectively, nursing education can benefit from enhanced family nursing skills development and provide students with valuable opportunities for practice and reflection.

Several studies have emphasized the significance of students' assumptions and beliefs regarding interactions with patients' families. In response, Happell and Gaskin [17] proposed that providing students with increased classroom education and longer clinical placements can foster a more positive attitude toward family assessment and the development of communication skills. Similarly, Tosterud et al. [18] suggested that simulation can be an effective method for teaching communication skills to nursing students at the basic level, enabling them to engage in effective conversations within a safe and virtual simulated clinical environment. Communication skills encompass a range of topics crucial to the development of nurses' clinical competence, particularly in the context of family assessment. This assessment is considered fundamental when students initiate patient interactions and interventions essential for achieving desired patient outcomes.

Additionally, Alammary [19] conducted a study examining the perception and satisfaction of novice nurses with high-fidelity simulation (HFS). The findings indicated that students had a highly positive perception of HFS, and its implementation facilitated nursing educators in providing feedback and training to novice students in areas where knowledge deficits were identified. This allowed students to engage in reflective practices, enhance their clinical thinking, and develop clinical judgment through specific learning activities. In contrast, Lindsey and Jenkins [20] argued that there is insufficient evidence to support the notion that HFSs effectively teach and enhance the skill of making sound clinical judgments. However, their study did not explore students' perceptions regarding the transferability of learning experiences from simulation to clinical practice.

Significance of the problem

Nursing students face the risk of inadequate clinical practice as a result of the limited availability of clinical teaching sites, reduced clinical hours, and a shortage of nursing faculty [2]. The proposed study is significant for several reasons. First, incorporating patients' families into treatment plans is essential for providing high-quality patient care and achieving positive patient outcomes. Therefore, it is important to identify effective educational approaches to enhance nursing students' ability to communicate and collaborate with their families. Second, simulation-based training has been shown to be an effective educational approach for enhancing nursing students' communication and collaboration skills. However, the effectiveness of simulation-based training in enhancing nursing students' perceptions of incorporating patients' families into treatment plans has not yet been well established. Therefore, the proposed study will help fill this gap in the literature by examining the effect of simulation-based training on nursing students' perceptions of incorporating patients' families into treatment plans. Finally, the findings of this study may have important implications for nursing education as they may inform the development of educational programs and curricula that better prepare nursing students for real-world clinical practice.

## Materials and methods

Aim of the study

This study aimed to investigate the effect of simulation training on enhancing nursing students' perception of integrating patients' families' assessments into their treatment plans. The following research objectives can be considered: (1) assess the baseline perceptions of nursing students regarding the importance of involving patients' families in their treatment plans. (2) Identifying the current practices of nursing students involving patients' families in their treatment plans. (3) Design and implement a simulation training program for nursing students to enhance their perceptions of involving patients' families in their treatment plans. (4) Evaluate the effectiveness of the simulation training program in enhancing nursing students' perceptions of involving patients' families in their treatment plans. (5) Propose recommendations to overcome the barriers to involving patients' families in their treatment plans, based on the study's findings. (6) To examine the association between students’ demographic backgrounds, perceptions, and family assessment skills.

Conceptual and theoretical framework of the study

**Figure 1:**
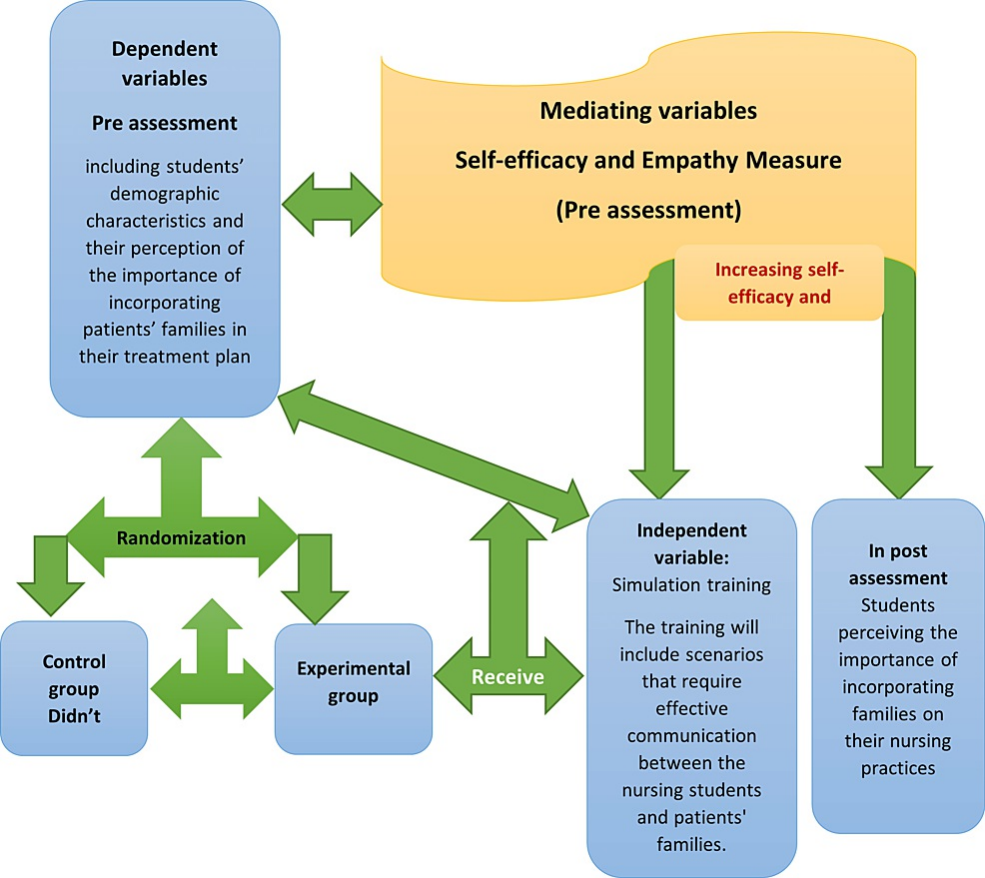
The conceptual framework of the simulation training study variables Developed by the 1st author (2023).

The theoretical framework for this investigation (Figure 1) suggests that simulation training can enhance nursing students' perceptions of the importance of incorporating patients' families into their treatment plans, with self-efficacy and empathy mediating this effect. Self-efficacy refers to nursing students' belief in their ability to incorporate patients' families into their treatment plans. Empathy refers to nursing students' ability to understand and share the feelings of patients and their families. Both self-efficacy and empathy were measured using a questionnaire administered to nursing students before and after simulation training.

On the other hand, simulation training's design is based on the theoretical framework, which comprises several theories, such as social learning theory, adult learning theory, communication theory, and patient-centered care concepts. Bandura's social learning theory [21] posits that individuals learn by observing others' behaviors and outcomes. In this research context, nursing students can learn about the significance of involving patients' families in treatment plans through simulations that demonstrate that healthcare professionals do so. Knowles [22] stressed that adults learn effectively when the learning material is applicable to their work, practical, and relevant. The simulation training in this study is intended to be pertinent and practical for nursing students because it replicates real-life situations that they may encounter in their future careers. Street Jr.'s communication theory [23] highlights the significance of clear and concise communication between healthcare providers and patients.

The simulation training in this study included scenarios that required effective communication between nursing students and patients' families. Patient-centered care, as defined by the Institute of Medicine [24], underscores the importance of integrating patients' values, preferences, and needs into their care. In the context of this study, involving patients' families in treatment plans is one way to provide patient-centered care.

Hypotheses

This study tested the following hypotheses.

Hypothesis 1

There is a statistically significant disparity in nursing students' perceptions of the importance of including patients' families in their treatment plans, before and after participating in the simulation training program.

Hypothesis 2

The simulation training program has a noteworthy impact on nursing students' inclusion of patients' families in their treatment plans.

Hypothesis 3

There is a notable correlation between nursing students' perception of obstacles to involving patients' families in their treatment plans and their perception of the significance of including families in the treatment plan.

Materials and methods

Study Design

A quantitative, experimental research design was used, with a control and intervention group using a pre- and post-test design to achieve the objectives of this study, i.e., to investigate the effect of simulation training on developing nursing students’ family assessment and communication skills for integrating patients' families on their treatment plan.

Study Subjects

In keeping with the experimental design, the study used a random sampling technique to allocate students into experimental and control groups. Students were randomly selected from the student information system and assigned to both the control and intervention groups. In the fall semester of the academic year 2020-2021, a cohort of 123 students out of a total of 160, spanning both the 7th and 8th academic levels, was included. This enrollment was specifically within senior nursing courses, including areas such as psychiatric nursing, pediatric and neonatology, maternity nursing, and community nursing. These courses necessitate the acquisition of skills in family assessment and communication. It was within this context that the inclusion criteria were established, requiring participants to meet the enrollment criteria and exhibit willingness to fully engage in all study sessions, encompassing pre-assessment, program implementation, and post-assessment.

The exclusion criteria were students who were in previous academic levels (4-6) and who were unwilling to participate. The Raosoft Sample Size Calculator (Raosoft Inc, Seattle, WA) was used to calculate the sample size with a margin of error of 5% and a confidence interval of 95%; hence, the minimum sample size was 114. The number of students was 123 undergraduate students out of 160 students, with consideration of students who would not accept to participate or who were on vacation or sick leave.

Tools of the Study

The data collection tools included the following three sections.

Section A: This section included a personal information form that was developed by the researchers after reviewing related literature and contained questions inquiring about the students (age, academic level, and experience with family).

Section B: Van Gelderen (2010) developed a rubric comprising 11 items related to communication, assessment, and integration of family-based care. The purpose of this rubric is to evaluate the family care and communication skills of nursing staff and students during simulation exercises, providing focused feedback during debriefing sessions. The title has been established as a reliable and valid tool for educators to enhance family care and communication skills among students and nursing staff. Each item in the title is assigned a score of three points for positive characteristics, two points for areas needing improvement, or one point for undesirable characteristics. The highest achievable score is 33/33 points, with a score range of 11-33.

Section C: Section C used in the study consists of 20 items and is conducted before and after the intervention. It utilizes a four-point Likert scale ranging from "not important" (1) to "very important" (4). The pre-survey (items one to nine) is administered to students during the first week of class, while the post-survey (items 10-20) utilizes a Likert scale ranging from "strongly agree" (4) to "strongly disagree" (1). The post-survey is distributed after students have observed faculty-led role-plays of patient-focused and family-focused assessments and have had the opportunity to practice their own family assessments. The authors of the study permit the free use of these scales for research and clinical purposes and have made them available in the public domain.

Validity and Reliability

The study tools have been tested for content validity and internal reliability in previous studies. Eleven of the 12 concepts exhibited significance at P = 0.05. Overall, the Van Gelderen Family Care Rubric (VGFCR) was determined reliably with Fleiss' kappa significance at P = 0.05 at the 95% confidence interval and Cronbach’s alpha = 0.842 among researchers and participants combined [25,26]. To ensure the validity and reliability of the tools, they were tested in the current study for content validity by experts and for reliability using Cronbach’s alpha correlation coefficient, which was reported as 95%, indicating that the rubric is highly reliable.

Data collection procedure

Simulation Program Content

Introduction: Simulation training on family involvement in client care was implemented for nursing students to provide hands-on practice and promote content mastery. The main principle of using simulation in nursing education is to replicate crucial features of a clinical situation so that students can learn in a non-threatening environment.

The main goal of this program was to change the scope of nursing practice by including the family as part of the assessment to ensure that the care of the patient is family-focused. Thus, the development of the program is the first to be conducted in three phases.

First Phase: Program Preparation

This phase was concerned with searching the literature, books, and research to prepare the theoretical and practical part of the program, which aimed to assist students in recognizing the benefits of having a family assessment in their clinical assessment of the patients; help inform and support nursing students about the importance of family inclusion in patient care and assessment; improvement in family assessment and communication skills of nursing students; enhance student learners’ learning experiences and help develop their clinical practice repertoires.

Second Phase: Program Implementation

Once the proposed study was approved by King Abdullah International Medical Research Center (KAIMRC) and the IRB, data collection was initiated after arrangement with the coordinators of nursing course levels 7 and 8. The students who agreed to participate in the study were asked to fill out a pre-survey and post-survey questionnaire about their perceptions of the family-based contents of the program.

The simulation learning is detailed as follows: students were introduced to the program through the use of observing two simulated role-plays. A recorded roleplay simulation scenario was used, including the principal investigator (PI) and two persons (one employee and faculty) who volunteered roles of the patients and a family member. The first simulated scenario exemplified the nurse’s interaction with the family in the hospital environment during an admission process focused on patient-centered care (the family is not even acknowledged). The second scenario focuses on using the family as a client-care approach (the family was invited to contribute) during the admission process.

The objective of this activity was to actively involve nursing students in developing skills that enable them to recognize and incorporate family-centered care. The remaining nursing students were assigned to critique the role of the nurse (PI) using the Van Gelderen simulation rubric (2010), which was published by Sophia University in 2012 after finalization [27]. During the debriefing session, these students provided feedback based on specific criteria, including communication style, positioning, eye contact, collection of family history and data, addressing family issues and concerns, use of medical jargon, nursing involvement, incorporating the family in the care of the hospitalized patient, addressing the needs of the family after hospitalization, offering support and hope, providing care based on a family-centered approach, and addressing family health routines.

During the debriefing session, nursing students were given the opportunity to elaborate on their critiques and highlight the contrasting differences between the two scenarios. Following this discussion, the students engaged in practicing their family assessment skills, taking on various roles such as nurses, patients, and family members. This experiential exercise allowed them to gain a fresh understanding and perspective by experiencing and embodying all these different roles.

The educational training program included 10 sessions conducted within two weeks, five sessions each week, and two sessions per day. One session covered the theoretical part of the simulation and the importance of family inclusion in patient care and assessment. Students were divided into small subgroups, and role-played scenarios showed that they gave feedback and their perception of the inclusion of family in patients’ assessment and care.

Methods of instructions: The sessions were presented to the participants with PowerPoint presentations on the family nursing theoretical background, recorded scripts and videos, written brochures, and audio-visual materials. Interactive lectures, brainstorming, roleplay, modeling, demonstration, re-demonstration discussion, and debriefing were used to teach the assessment of family inclusion using communication skills and interventions for certain client scenarios.

Third-Phase: Post-program Evaluation

Following the simulation experience, the PI compared the data and videotaped recordings, which helped to see and evaluate student performance and content knowledge of family-based care. Finally, the PI and the coauthor viewed the videotapes of the student nurses as they made family assessments and evaluated student performance using the Van Gelderen simulation rubric [27]. Research reliability and rigor were maintained by two educators who independently evaluated the nursing students’ family assessment techniques. They consisted of the PI and the coauthor who were PhD-prepared nurse educators with current nursing clinical practice backgrounds.

Data collection procedure

The data collection procedure used in this study involved several steps. First, the simulation sessions were developed and reviewed by expert academic members and a rubric was designed to assess students' learning outcomes. Second, an electronic pretest questionnaire was administered to both the experimental and control groups in week two of the fall semester. The questionnaire focused on simulating effective communication and the assessment of family needs using the VGFCR. Next, the intervention group participated in a recorded role-play simulation session conducted by faculty members, which involved effective communication and the assessment of family needs. The simulation was delivered in-person (for level 8 students) and virtually, considering the feasibility during the data collection period. By contrast, the control group did not participate in this session.

Subsequently, both groups were asked to complete an electronic post-test questionnaire using the same rubric at week eight, along with post-survey questionnaires. It is important to note that the content of the simulation session did not impact the students' assessments, and the control group was not exposed to the intervention at week nine to ensure the standardization of content.

Data management and analysis plan

Data were coded and analyzed using SPSS (IBM Corp., Armonk, NY). Data were presented using descriptive statistics for discrete variables in the form of frequencies and percentages, means, and standard deviations. Chi-square, Fisher’s exact test, and paired t-test were used to analyze the total scores of the participant's responses on the pre-test and post-test (i.e., before and after the simulation training). For comparisons between numerical data, Student’s t-test and analysis of variance (ANOVA) were applied to identify the correlation value between the studied variables. The significance level was adjusted and tested at p < 0.05.

Ethical considerations

The study was approved by the research unit at the College of Nursing, Jeddah, KAIMRC, and the IRB. Subsequently, the study subjects were approached to explain the purposes and procedure of the study. The subjects were informed that their participation in the study was voluntary and that they could withdraw without any penalty at any time. They were assured that their answers would be kept anonymous during the study and that their data would be kept confidential. The PI ensured that all data, both hard and soft copies, were stored within the Ministry of National Guard Health Affairs (MNGHA) premises and accessed by the research team only.

## Results

Table 1 provides information on the age, academic level, marital status, and parental status of the studied students. The age of the participants ranged from 19 to 23 years, with a mean age of 21.3 ± 1.3 in the control group and 22.2 ± 1.1 in the experimental group. The majority of the control group (83.9%) was at the seventh academic level, while 70.1% of the experimental group were at the same level. In terms of marital status, most students in both groups were single, accounting for 94.6% in the control group and 88.1% in the experimental group. Among the married students, only one out of three in the control group reported having children, compared to four out of eight in the experimental group. It is worth noting that there were no statistically significant associations observed between the two groups in these characteristics.

**Table 1:** Distribution of the studied groups (control and experimental groups) according to their personal characteristics (N = 123) X2: chi-square test; FET: Fisher's exact test; P: P-value of test of significance.

Personal characteristics	Control group		Experimental group		Sig.
	No.	%	No.	%	
Age (years)					
19-20	9	16.1	4	6.0	FET: 3.985; P: 0.136
21-22	32	57.1	38	56.7	
23 and more	15	26.8	25	37.3	
Mean ± SD	21.3 ± 1.3		22.2 ± 1.1		
Academic level					
Level 7	47	83.9	47	70.1	X^2^: 3.214; P: 0.090
Level 8	9	16.1	20	29.9	
Marital status					
Single	53	94.6	59	88.1	FET: 2.357; P: 0.502
Married	3	5.4	6	9.0	
Divorced	0	0.0	1	1.5	
Widow	0	0.0	1	1.5	
Have children	n = 3		n = 8		
Yes	1	33.3	4	50.0	FET: 0.244; P: 0.621
No	2	66.7	4	50.0	

Table 2 presents several findings related to students' experiences and perceptions of family assessment. Approximately half of the students in both the control group (44.6%) and experimental group (53.7%) had previous experience in family assessment. Regarding sources of information about family assessment, approximately 60.7% of the control group reported getting their information from lectures and workshops, while slightly over half (50.7%) of the experimental group obtained their information from similar sources.

**Table 2:** Distribution of the studied groups (control and experimental groups) according to their previous experience in family care (N = 123) X2: chi-square test; FET: Fisher's exact test; P: P-value of test of significance.

Variables	Control group		Experimental group		Sig.
	No.	%	No.	%	
Previous experience with family assessment in client care?					
No	31	55.4	31	46.3	X^2^: 1.008; P: 0.315
Yes	25	44.6	36	53.7	
Family assessment-related source of information					
Lectures	19	33.9	33	49.3	FET: 5.402; P: 0.092
Workshops	3	5.4	0	0.0	
Mixed	34	60.7	34	50.7	
Working experience					
No	44	78.6	50	74.6	X^2^: 0.263; P: 0.608
Yes	12	21.4	17	25.4	
Currently use family assessment in dealing with clients					
No	27	48.2	27	40.3	X^2^: 4.984; P: 0.083
Yes	8	14.3	21	31.3	
Sometimes	21	37.5	19	28.4	
I have been admitted as a patient in a healthcare setting					
No	19	33.9	22	32.8	X^2^: 0.016; P: 0.898
Yes	37	66.1	45	67.2	
I felt my family members were respected and included in my care	n = 37		n = 45		
Strongly disagree	2	5.4	1	2.2	FET: 4.135; P: 0.243
Disagree	3	8.1	2	4.4	
Agree	15	40.5	28	62.2	
Strongly agree	17	45.9	14	31.1	
I have been a family member of a patient in a healthcare setting	n = 56		n = 67		
No	20	35.7	18	26.9	X^2^: 1.119; P: 0.290
Yes	36	64.3	49	73.1	
How comfortable are you in working with families in a healthcare setting					
Very uncomfortable	1	2.8	1	2.0	FET: 0.706; P: 0.929
Uncomfortable	5	13.9	5	10.2	
Comfortable	19	52.8	28	57.1	
Very comfortable	11	30.6	15	30.6	

Approximately one-quarter of the participants in the control group (21.4%) and the experimental group (25.4%) had work experience. More than half of the students in both the control group (51.8%) and the experimental group (59.7%) currently use family assessment in their interactions with clients.

Furthermore, approximately two-thirds of the students in both the control group (66.1%) and experimental group (67.2%) were admitted as patients in healthcare settings. Among those previously admitted to the hospital in the control group, 45.9% strongly agreed that they felt their family members were respected and included in their care, whereas this percentage was 31.1% in the experimental group. Moreover, 64.3% of the control group reported working with different families in healthcare settings compared to 73.1% of the experimental group.

In terms of comfort levels in working with families in healthcare settings, more than half of the students in both the control group (52.8%) and the experimental group (57.1%) expressed feeling comfortable with this type of care. Importantly, no statistically significant associations were noted between the two groups.

Table 3 presents the students’ perceptions of the importance of family care. Overall, the table indicates that there was no statistically significant difference between the control group's perception in the pre- and post-training phases (first and second assessment) regarding various aspects of family care. These aspects include understanding the family's beliefs about health care, interacting with families in a healthcare setting, collecting family history during patient admission, addressing family issues and concerns during patient admission, addressing the need for follow-up care during assessment at admission, offering support and hope to the family, addressing family health routines, and addressing ethical and social justice inequities within family units.

**Table 3:** Distribution of the studied groups (control and experimental groups) according to their perception of the importance of family care (N = 123) P1: Significance comparison between the control group (pre and post). P2: Significance comparison between the study groups (pre and post). P3: Significance comparison between the control and experimental groups (pre). P4: Significance comparison between the control and experimental groups (post). *: Significance at P ≤ 0.05.

Students perceived importance in	Control group		Sig. t-test (P1)	Experimental group		Sig. t-test (P2)	Sig. t-test (P3)	Sig. t-test (P4)		
	Mean ± SD			Mean ± SD						
	Pre	Post		Pre	Post					
How important is it to include family members as part of the care of the patient?	2.4 ± 0.7	2.5 ± 0.7	t: -2.803; P1: 0.007*	2.4 ± 0.8	2.8 ± 0.3	t: -3.460; P2: 0.001*	t: 0.009; P3: 0.993		t: -2.306; P4: 0.023*	
How important is it to understand the family’s beliefs about health care?	2.5 ± 0.6	2.5 ± 0.6	t: -1.137; P1: 0.261	2.4 ± 0.7	2.7 ± 0.4	t: -3.616; P2: 0.001*	t: 0.534; P3: 0.595	t: -2.442; P4: 0.016*		
How important is it for the nurse to interact with families in a healthcare setting?	2.6 ± 0.5	2.6 ± 0.5	t: 0.000; P1: 1.000	2.4 ± 0.7	2.8 ± 0.3	t: -3.389; P2: 0.001*	t: 1.247; P3: 0.215	t: -2.331; P4: 0.021*		
How important is it for the nurse to collect family history during a patient admission?	2.7 ± 0.5	2.7 ± 0.5	t: 1.427; P1: 0.159	2.5 ± 0.7	2.9 ± 0.2	t: -3.808; P2: <0.001*	t: 1.828; P3: 0.070	t: -2.389; P4: 0.018*		
How important is it for the nurse to address family issues and concerns during a patient's admission?	2.3 ± 0.7	2.3 ± 0.7	-	2.2 ± 0.7	2.6 ± 0.4	t: -4.564; P2: <0.001	t: 1.009; P3: 0.315	t: -2.937; P4: 0.004*		
How important is it for the nurse to address the need for follow-up care during an admission assessment?	2.4 ± 0.7	2.3 ± 0.7	t: 1.000; P1: 0.322	2.3 ± 0.7	2.7 ± 0.4	t: -3.986; P2: <0.001*	t: 0.502; P3: 0.617	t: -3.253; P4: 0.001*		
How important is it to offer support and hope to the family?	2.4 ± 0.7	2.4 ± 0.7	t: 1.000; P1: 0.322	2.4 ± 0.6	2.7 ± 0.5	t: -2.825; P2: 0.006*	t: 0.503; P3: 0.616	t: -2.378; P4: 0.019*		
How important is it for the nurse to address family health routines?	2.1 ± 0.8	2.1 ± 0.8	t: 1.000; P1: 0.322	2.3 ± 0.8	2.4 ± 0.5	t: -1.155; P2: 0.252	t: 1.313; P3: 0.192	t: -2.769; P4: 0.007*		
How important is it for the nurse to address ethical and social justice inequities within family units?	2.4 ± 0.7	2.4 ± 0.7	t: 1.000; P1: 0.322	2.3 ± 0.8	2.7 ± 0.4	t: -3.334; P2: 0.001*	t: 1.129; P3: 0.261	t: -2.126; P4: 0.036*		

However, a statistically significant difference was observed in their perception of the importance of including family members in a patient's care, with a t-value of -2.803 and a p-value of 0.007. Furthermore, in the experimental group, there was an improvement in the mean scores in the post-training phase compared to the pre-training phase. For all items related to their perception of the importance of family care, this improvement was statistically significant at a p-value of less than 0.05. Notably, there were no significant differences between the control and experimental groups in the pretraining phase. However, in the post-training phase, a statistically significant difference was observed between the two groups in all items related to their perceptions of the importance of family care.

Table 4 presents the results, indicating a statistically significant difference in the mean scores between the experimental and control groups regarding their perception of the importance of simulation as an effective tool for learning family care. The p-value was less than 0.05 for the following aspects: (1) the importance of using two nurse-family simulation roles is to enhance the understanding of family as client care. (2) The importance of simulation debriefing time during the learning process. (3) The importance of taking the opportunity to practice family-focused care assessments in nursing laboratories. (4) The importance of taking the opportunity to play the role of a family member during practice. (5) It is important to understand and feel that the use of family genograms in clinical practice is important. (6) Understanding and feeling that family ecomaps are used in the clinical practice environment is important. (7) The importance of learning more about family as client care. (8) The importance of role-plays in enhancing knowledge of ethical and social justice inequities within family units. (9) The importance of recommending this simulated family assessment experience to future nursing students.

**Table 4:** Distribution of the studied groups (control and experimental groups) according to their perception of simulation as an effective learning tool for family care (N = 123) *: Significance at P ≤ 0.05.

Students perceived importance in	Group		Sig.
	Control group	Experimental group	
	Mean ± SD	Mean ± SD	
1. I felt the two nurse-family simulation role-plays contributed to my understanding of family as client care.	2.1 ± 0.7	2.3 ± 0.6	t: 2.878; P: 0.005*
2. The simulation debriefing time (time spent talking about the scenarios) was beneficial to my learning.	2.2 ± 0.7	2.7 ± 0.4	t: -3.989; P: <0.001*
3. Having the opportunity to practice family-focused care assessments in the nursing lab was important to me.	2.1 ± 0.8	2.5 ± 0.5	t: -3.240; P: 0.002*
4. Having the opportunity to play the role of a family member during practice time was an important piece of my learning about family members’ feelings.	2.0 ± 0.7	2.3 ± 0.5	t: -2.068; P: 0.041*
5. I understand the use of family genograms in the clinical practice environment.	2.3 ± 0.7	2.5 ± 0.4	t: -2.064; P: 0.041*
6. I feel the use of family genograms in the clinical practice environment is important.	2.5 ± 0.5	2.6 ± 0.4	t: -2.126; P: 0.036*
7. I understand the use of family ecomaps in the clinical practice environment.	2.2 ± 0.6	2.5 ± 0.5	t: -2.810; P: 0.006*
8. I feel the use of family ecomaps in the clinical practice environment is important.	2.2 ± 0.5	2.4 ± 0.5	t: -2.284; P: 0.024*
9. Learning more about family as client care is important to me.	1.9 ± 0.8	2.3 ± 0.7	t: -2.517; P: 0.013*
10. The role-plays enhanced my knowledge of ethical and social justice inequities within family units.	2.3 ± 0.7	2.6 ± 0.7	t: -2.685; P: 0.008*
11. I would recommend this simulated family assessment experience for future nursing students.	2.3 ± 0.7	2.6 ± 0.7	t: -2.327; P: 0.022*

In summary, Table 4 demonstrates that the experimental group had significantly higher mean scores than the control group, indicating their stronger perception of the importance of simulation in various aspects of learning about family care.

Table 5 presented the experimental group’s evaluation of their family assessment simulation experience in either scene one or two by using the Van Gelderen simulation rubric. Eleven domains of the simulation rubrics were included. There was a statistically significant difference noted between scenes one and two (p-value less than 0.05) in the nurse communication style domain, the use of terminology, nurse positioning and addressing needs for follow-up care domains, the nurse’s eye contact domain, family history and data collection method domain, addressing family issues and concerns domain, addressing nursing involvement domain, offering support and hope as one of the evaluation domains, and their evaluation of the family as a client care approach and family health routines domain.

**Table 5:** Distribution of the experimental group according to their evaluation of the family assessment simulation experience (Scenes 1 and 2) using the Van Gelderen simulation rubric (2010) F: ANOVA test; P: P-value of test of significance; *: Significance at P ≤ 0.05.

Simulation evaluation	Evaluation of Scene 1		Evaluation of Scene 2		Sig.
	No.	%	No.	%	
1. Nurse communication style					
Undesirable characteristics	22	32.8	0	0.0	F: 7.761; P: 0.001*
Characteristics needing improvement	37	55.2	13	19.4	
Positive characteristics	8	11.9	54	80.6	
Mean ± SD	1.7 ± 0.6		2.8 ± 0.3		
2. Use of terminology					
Undesirable characteristics	19	28.4	0	0.0	---------
Characteristics needing improvement	40	59.7	0	0.0	
Positive characteristics	8	11.9	67	100.0	
Mean ± SD	1.8 ± 0.6		3.0 ± 0.0		
3. Nurse positioning					
Undesirable characteristics	15	22.4	0	0.0	---------
Characteristics needing improvement	44	65.7	0	0.0	
Positive characteristics	8	11.9	67	100.0	
Mean ± SD	1.8 ± 0.5		3.0 ± 0.0		
4. Nurse eye contact					
Undesirable characteristics	5	7.5	0	0.0	F: 4.967; P: 0.010*
Characteristics needing improvement	19	28.4	13	19.4	
Positive characteristics	43	64.2	54	80.6	
Mean ± SD	2.5 ± 0.6		2.8 ± 0.3		
5. Family history and data collection method					
Undesirable characteristics	46	68.7	13	19.4	F: 142.419; P: <0.001*
Characteristics needing improvement	13	19.4	10	14.9	
Positive characteristics	8	11.9	44	65.7	
Mean ± SD	1.4 ± 0.7		2.4 ± 0.8		
6. Addressing family issues and concerns					
Undesirable characteristics	41	61.2	13	19.4	F: 60.848; P: <0.001*
Characteristics needing improvement	18	26.9	0	0.0	
Positive characteristics	8	11.9	54	80.6	
Mean ± SD	1.5 ± 0.7		2.6 ± 0.7		
7. Addressing nursing involvement					
Undesirable characteristics	29	43.3	13	19.4	F: 21.731; P: <0.001*
Characteristics needing improvement	25	37.3	0	0.0	
Positive characteristics	13	19.4	54	80.6	
Mean ± SD	1.7 ± 0.7		2.6 ± 0.7		
8. Addressing needs for follow-up care					
Undesirable characteristics	41	61.2	0	0.0	--------
Characteristics needing improvement	18	26.9	0	0.0	
Positive characteristics	8	11.9	67	100.0	
Mean ± SD	1.5 ± 0.7		3.0 ± 0.0		
9. Offer of support and hope					
Undesirable characteristics	41	61.2	0	0.0	F: 60.848; P: <0.001*
Characteristics needing improvement	18	26.9	13	19.4	
Positive characteristics	8	11.9	54	80.6	
Mean ± SD	1.5 ± 0.7		2.8 ± 0.3		
10. Provided care based upon the "Family as Client" approach					
Undesirable characteristics	46	68.7	0	0.0	F: 72.519; P: <0.001*
Characteristics needing improvement	0	0.0	13	19.4	
Positive characteristics	21	31.3	54	80.6	
Mean ± SD	1.6 ± 0.9		2.8 ± 0.3		
11. Family health routines are assessed					
Undesirable characteristics	32	47.8	0	0.0	F: 35.701; P: <0.001*
Characteristics needing improvement	14	20.9	13	19.4	
Positive characteristics	21	31.3	54	80.6	
Mean ± SD	1.8 ± 0.8		2.8 ± 0.3		

Table 6 displays the Van Gelderen simulation rubric (2010), which was utilized to evaluate students’ performance in a family assessment role-play exercise following family assessment simulation sessions. The rubric includes 11 assessment domains. The majority of students exhibited positive attributes in various areas, such as communication style, maintaining eye contact, addressing family issues and concerns, acknowledging nursing involvement, offering support and hope, adopting a family-as-client approach, and assessing family health routines. Each of these domains received an assessment score of 81.3%.

**Table 6:** Student’s family assessment role-play simulation evaluation rubric (Van Gelderen simulation rubric, 2010) for the researcher evaluation of student’s family assessment experience post-role-play using the rubric

Van Gelderen simulation rubric (2010)	No.	%
1. Nurse communication style		
Undesirable characteristics	0	0.0
Characteristics needing improvement	3	18.8
Positive characteristics	13	81.3
Mean ± SD	2.8 ± 0.4	
2. Use of terminology		
Undesirable characteristics	0	0.0
Characteristics needing improvement	0	0.0
Positive characteristics	16	100.0
Mean ± SD	3.0 ± 0.0	
3. Nurse positioning		
Undesirable characteristics	0	0.0
Characteristics needing improvement	0	0.0
Positive characteristics	16	100.0
Mean ± SD	3.0 ± 0.0	
4. Nurse eye contact		
Undesirable characteristics	0	0.0
Characteristics needing improvement	3	18.8
Positive characteristics	13	81.3
Mean ± SD	2.8 ± 0.4	
5. Family history and data collection method		
Undesirable characteristics	3	18.8
Characteristics needing improvement	2	12.5
Positive characteristics	11	68.8
Mean ± SD	2.5 ± 0.8	
6. Addressing family issues and concerns		
Undesirable characteristics	3	18.8
Characteristics needing improvement	0	0.0
Positive characteristics	13	81.3
Mean ± SD	2.6 ± 0.8	
7. Addressing nursing involvement		
Undesirable characteristics	3	18.8
Characteristics needing improvement	0	0.0
Positive characteristics	13	81.3
Mean ± SD	2.6 ± 0.8	
8. Addressing needs for follow-up care		
Undesirable characteristics	0	0.0
Characteristics needing improvement	0	0.0
Positive characteristics	16	100.0
Mean ± SD	3.0 ± 0.0	
9. Offer of support and hope		
Undesirable characteristics	0	0.0
Characteristics needing improvement	3	18.8
Positive characteristics	13	81.3
Mean ± SD	2.8 ± 0.4	
10. Provided care based upon the "Family as Client" approach		
Undesirable characteristics	0	0.0
Characteristics needing improvement	3	18.8
Positive characteristics	13	81.3
Mean ± SD	2.8 ± 0.4	
11. Family health routines are assessed		
Undesirable characteristics	0	0.0
Characteristics needing improvement	3	18.8
Positive characteristics	13	81.3
Mean ± SD	2.81 ± 0.4	

Additionally, all students demonstrated positive characteristics in their use of appropriate terminology, maintaining proper positioning during the assessment process, and recognizing the need for follow-up care. Furthermore, over two-thirds (68.8%) of students displayed positive characteristics in their assessment of family history and data collection methods.

**Figure 2:**
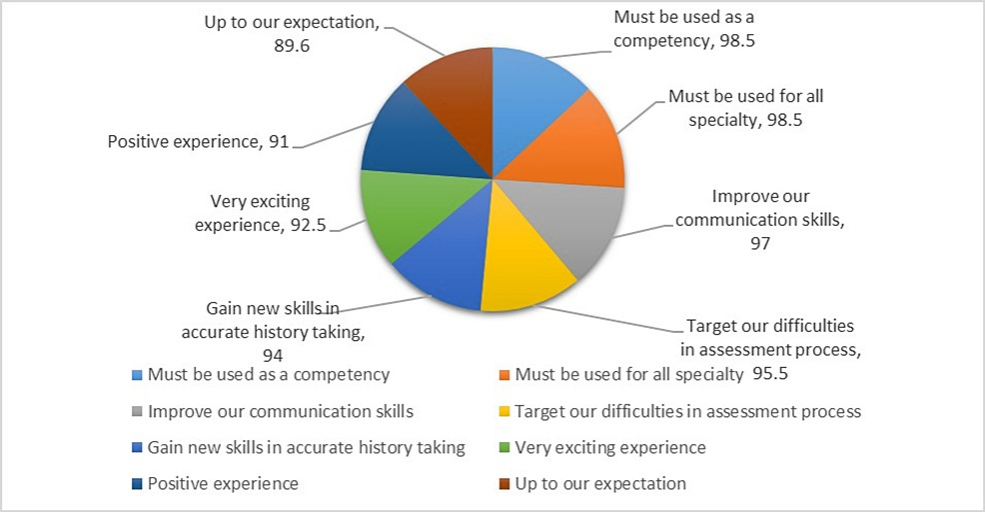
Post-debriefing experimental group feedback regarding simulation

Figure 2 depicts the feedback provided by the experimental group in the post-debriefing session regarding their experiences with the family assessment simulation. The overwhelming majority of students (98.5%) expressed their opinion that family assessment simulation should be incorporated as competency in various courses, including community, psychiatric, pediatric, obstetric, and critical courses, among others. Furthermore, most students believed that participating in the simulation enhanced their communication skills and helped them address the difficulties encountered during the assessment process. Additionally, they reported acquiring new skills in conducting accurate history taking, with 97%, 95.5%, and 94% of students acknowledging these improvements. Finally, the majority of students (92.5%, 91%, and 89.6%) shared that the simulation experience was highly exciting, positive, and met their expectations.

## Discussion

Simulation-based learning offers a wide range of opportunities to practice complex skills in higher education and implement different types of scaffolding to facilitate effective learning [6]. With the increasing difficulty of securing clinical placements in Bachelor of Nursing programs, the use of simulation as a learning enhancement tool is becoming more prevalent. Simulation videos integrated into a blended learning approach offer students the opportunity to observe and learn from exemplary practices, while also establishing meaningful connections between theoretical knowledge and its practical application [28].

The strength of this study lies in that it is the first study to focus on the perception of nursing students regarding the participation of families in the care of their patients. To determine the effectiveness of the simulation nursing program as a strategy to develop undergraduate nursing students to integrate patients’ families into their treatment plan, an online questionnaire was administered, and 114 (71.3%) out of 160 undergraduate nursing students at the 7-8th level answered it. Hernández-Martínez et al. [27] observed a comparable rate of 70.1%, mirroring the findings in their study that elucidated the encounters and viewpoints of nursing university degree students engaged in the role of health support amid the COVID-19 health crisis. Therefore, the current study aimed to investigate the effect of simulation training on developing nursing students' perceptions of integrating patients' families as assessments of their treatment plans.

The students who were engaged in the current study were aged between 19 and 23 years and 23 years and above, with a mean age of 21.3 ± 1.3 years among the control group and 22.2 ± 1.1 years among the experimental group. Most of the control group was in the seventh academic level, compared to 70% of the experimental group. Most of the students in both the control and experimental groups were married. These results support those of Joseph et al. [29], who studied the perception of simulation-based learning among medical students in South India in the same age groups as well as at the same academic level.

Regarding the students’ previous experience in family assessment, the current study showed that around half of the students in both the control and experimental groups had previous experience in family assessment. More than half of the students in the control and experimental groups used family assessment to deal with clients. Approximately two-thirds of the students in the control and experimental groups were admitted to the healthcare setting. Among those of the control group who were previously admitted to the hospital, less than half declared that they strongly agreed that their family members were respected and included in their care, compared to 30% of the experimental group. Furthermore, more than two-thirds of the control group reported that they worked with different families in healthcare settings compared to less than three-quarters of the experimental group. Regarding the degree of comfort in working with families in healthcare settings, more than half of the control and experimental groups reported feeling comfortable with this type of care.

Nearly the same findings were reported by Cené et al. [28], who stated that patient and family engagement (PFE) is vital to the spirit of the medical home. Although PFE appeals to patients, families, providers, and policymakers, research is needed to assess outcomes beyond satisfaction, address implementation barriers, and support. Additionally, family-focused care and communication are recognized as the best practice when caring for patients and families, as Van Gelderen et al. [30] suggested to improve healthcare outcomes from Christian [31] and Mann [32].

Involving family members in the medical care of patients is often preferred by patients themselves, as family members can provide valuable information about the patient's functioning at home and support treatment compliance. However, Shibily et al. [33] conducted a study on nurses and nursing students and found that nurses with more years of experience showed less support for family involvement in patient care. This negative correlation may be attributed to the time constraints experienced by senior nurses who are expected to multitask.

Family involvement plays a crucial role in patient-centered care and has a significant impact on the quality of care and patient outcomes. Jazieh et al. [34] proposed a communication model that emphasizes structured communication with the family while keeping the patient at the center. This model involves identifying the most responsible family member and respecting the patient's autonomy and rights. Implementing this communication model can facilitate family involvement, particularly in societies with large families.

While the healthcare environment primarily focuses on patient care, an increasing number of researchers argue that family-centered care leads to improved health outcomes and cost reduction. Hernández-Martinez et al. [27] highlight the importance of understanding students' perceptions of family care. Their study found no significant difference in the control group's perception of various aspects of family care before and after training, except for the importance of including family members in a patient's care. These findings are consistent with those of Joseph et al. [29]. Kokorelias et al. [35] also support the notion that family-centered care models enhance communication and the exchange of information among family members, patients, and healthcare providers, ultimately contributing to the development and delivery of effective care plans.

The recent study demonstrated a significant improvement in the mean scores of the experimental group's perceptions of the importance of family care during the post-training phase compared to the pre-training phase. This improvement was statistically significant (p < 0.05) across all items. However, there were no significant differences observed between the control and experimental groups during the pre-training phase. In contrast, a statistically significant difference emerged between the two groups in the post-training phase across all items related to the perception of the importance of family care. These findings reaffirm that families can exert influence on health outcomes through various pathways, including biological, behavioral, and psychophysiological factors. Biological pathways involve the transmission of infectious agents, exposure to similar toxic environments, and genetic vulnerabilities. Health behavior pathways encompass lifestyle choices like smoking, exercise, diet, and substance abuse. Additionally, healthcare behaviors, such as adherence to treatment and family caregiving, play a role. Pathophysiological pathways refer to the impact of the family environment on neuroendocrine and immunological processes [36-39]. To promote a provider-family relationship that comprehends and addresses the needs of patients and their families, family assessment should be an integral part of each patient's assessment [40]. Elcokany and Abdel Wareth [39] emphasize the necessity of establishing policies, procedures, and protocols for family engagement in intensive care units (ICUs). Furthermore, there should be an assessment tool available to determine the willingness of families to actively participate in patient care and to identify specific aspects of care in which they can be involved.

The current study also highlighted the students' perceptions of simulation as an effective tool for learning family care. Significant differences were observed between the experimental and control groups in various aspects, indicating the importance of simulation in enhancing their understanding and skills. These aspects include the use of nurse-family simulation roles, simulation debriefing time, practicing family-focused care assessments, role-playing as family members, using family genograms and ecomaps in the clinical practice environment, learning about family as client care, understanding ethical and social justice issues within family units, and recommending simulated family assessment experiences to future nursing students. The researchers emphasized the value of professionally developed videos to ensure consistency and facilitate concept review, thereby improving educational standards.

These findings are consistent with previous research in the field. Joseph et al. [29] reported that participants in their study had favorable perceptions of simulation-based learning (SBL), particularly among female students and senior students in later semesters. The majority of students agreed that simulation supported the development of clinical skills, and a significant portion expressed openness to replacing real patients with simulated patients in practical examinations. Joseph et al. [29] concluded that SBL was viewed positively by participants, suggesting its potential implementation in medical curricula. Similarly, Veltri et al. [40] found supportive results for replacing traditional clinical experiences with simulation, as it allows for objective evaluation and benchmarking of clinical reasoning capabilities beyond simple pass/fail assessments of student competency.

Similarly, Al Enazi [41] conducted a study to assess students' perceptions of simulation in nursing education. The findings indicated that students highly valued the simulation experience and believed it should be an integral part of their clinical training. Simulation debriefing sessions were particularly helpful in enhancing students' understanding and reasoning. The study also found that clinical experience did not significantly influence students' perceptions of simulation, but female students reported experiencing more nervousness during simulations compared to male students. Additionally, students with prior simulation experience perceived simulations as more realistic than those without previous exposure.

In addition to recognizing the significance of involving families as consumers, it is essential for teachers to acknowledge the valuable insights and unique perspectives that families can offer. Rutland and Hall [42] emphasized the role of teachers in promoting the active participation of families in the assessment process, encouraging them to serve as informants, team members, advocates, and consumers. On the other hand, Kim et al. [43] underscored the importance of enhancing virtual simulation for prelicensure nursing students to improve their confidence and competence. This involves addressing areas such as increasing realism, enhancing engagement, and maximizing user satisfaction and performance.

Hoper's [44] study explored the use of simulation in enhancing family assessment experiences among students and identified three themes: conceptualizing the learning experience, capturing the big picture, and connecting with the team. Notably, the VGFCR was developed to improve students' family care and communication skills in simulation. Van Gelderen et al. [25] demonstrated the reliability and validity of the VGFCR as a tool to assist educators in developing students' and nursing staff's family care and communication skills.

In the current study, the experimental groups evaluated their family assessment simulation experience using the Van Gelderen simulation rubric, which consists of 11 domains. Significant differences were observed between scene one and scene two, particularly in the domains of nurse communication style, use of terminology, nurse positioning, addressing needs for follow-up care, nurse's eye contact, family history and data collection methods, addressing family issues and concerns, addressing nursing involvement, offering support and hope, and family as a client care approach and family health routines. Similarly, Seo et al. [45], in their study on the effect of simulation nursing education on clinical reasoning, problem-solving, self-efficacy, and clinical competency in Korean nursing students, found that the experimental group achieved significantly higher scores in clinical competency compared to the control group.

The growing utilization of simulation in nursing education has created a need for reliable and valid evaluation tools to assess student learning. Educational rubrics offer predefined criteria and expectations that educators can use to evaluate students' competence and provide feedback. However, Adamson et al. [46] found that existing simulation evaluation instruments did not focus on family care. While the Creighton Simulation Evaluation Instrument (C-SEI) emphasized communication skills, it did not measure family communication. In contrast, Van Gelderen et al. [25] highlighted the significance of their rubric in developing family care and communication skills among nursing staff and students. A helpful tool for educators, the VGFCR, makes it easier to teach and make improvements to family-focused care actions while also aiding students' comprehension and skill development in family care and communication in simulation.

In the current study, the Van Gelderen simulation rubric [47] was employed to evaluate students' family assessment role-play experience during family assessment simulation sessions. The rubric encompassed 11 assessment domains. The majority of students exhibited positive characteristics in domains such as communication style, eye contact, addressing family issues and concerns, nursing involvement, offering support and hope, using a family as a client approach, and assessing family health routines, with over 80% proficiency in each domain. Moreover, all students demonstrated positive attributes in using appropriate terminology, positioning during assessment, and addressing the need for follow-up care. Additionally, more than two-thirds of students exhibited positive characteristics in assessing family history and data collection methods. This aligns with Mohamed and Fashafsheh's [5] findings, which showed that simulation-based training resulted in significant improvement in communication skills, self-efficacy, and clinical competence among participants. Furthermore, there was a significant relationship between gender and clinical competency. These findings support the effectiveness of simulation-based training in enhancing communication skills, self-efficacy, and clinical competence, suggesting that incorporating multiple-patient simulations in nursing curricula is highly recommended. Additionally, Van Gelderen [47] observed a trend of increasing the perceived importance of family care among students after their involvement in simulations, although statistical significance was not found. The implementation of simulation role-play for undergraduate and sophomore nursing students to enhance family communication and assessment skills was positively perceived, and replication of this simulation experience was recommended for future nursing students.

Another relevant study by van den Bos-Boon et al. [48] examined the effectiveness of simulation training and assessment of pediatric intensive care unit (PICU) nurses' resuscitation skills. They reported improvements in communication skills, increased self-confidence in resuscitation skills, and decreased dependency on physicians among nurses. Proficiency checks enhanced nurses' confidence and empowered them to initiate resuscitation promptly after a child's collapse. Most nurses believed that the time taken to initiate resuscitation had decreased, indicating the positive impact of simulation training on their skills and confidence.

Regarding the post-debriefing feedback from the experimental group in the current study, the majority of students expressed that family assessment simulation should be included as a competency in their education across various courses. They believed that the experience improved their communication skills, addressed difficulties in the assessment process, and provided new skills in accurate history taking. Additionally, students found the simulation experience exciting, positive, and in line with their expectations. The study aimed to determine the percentage of students who preferred simulation over direct contact with infected patients, recognizing the risks nursing students face in hospitals without having completed their degree. This experience may have also negatively impacted healthcare workers, who faced high stress due to the increased risk of infection [7,48,49]. Furthermore, simulation training demonstrated better preparedness scores compared to exclusively theoretical training. These findings align with previous studies that have shown the benefits of simulation in preparing students for real-world nursing practice. It is noteworthy that both groups in the study had experience with family assessments as part of their current practice, indicating the relevance and applicability of simulation-based training in the context of family care.

Recommendations and implications

It is essential to encourage nursing students to adopt family-centered care and involve family members in the healthcare process. Collaboration among patients, medical staff, and families is crucial for effective family-centered care.

This study's strengths include the use of standardized scenarios and a validated measurement tool to assess communication skills when integrating family members. Incorporating simulation-based assessments in nursing curricula can greatly contribute to family assessment training.

The study supports the effectiveness of standardized patient simulation and scenario-based learning in bridging the gap between theory and practice. Publishing the benefits of simulation in family assessment training can further enhance students' skills in addressing diverse patient needs and concerns.

The findings suggest the need to revise nursing curricula to include family assessment and employ evidence-based strategies such as simulation technology to close the theory-practice gap for graduates entering clinical practice.

Effective learning activities should closely resemble real-world patient-care environments, match students' knowledge levels, involve trained faculty, and include debriefing sessions to promote active and engaging learning.

Simulation's extensive impact includes improved critical thinking, clinical judgment, and the ability to apply theoretical concepts in practice, leading to enhanced patient safety and outcomes identifying barriers to nurses' support for patient- and family-centered care, such as hospital policies and integration models, can facilitate better communication between nurses and families.

Future research directions should explore the impact of simulation training on patient outcomes related to proficiency tests. It is crucial to assess whether improved assessment skills lead to better patient outcomes, considering the various factors that may influence results.

Further investigation is needed to determine whether nurses experience reduced stress and negative emotions as they gain more experience with assessments. Informing nursing students about the assessment process and reassuring them that poor performance has no consequences could help alleviate assessment-related stress.

## Conclusions

The findings of the study indicated that the utilization of scenario-based SP-simulated exercises, guided by dedicated faculty and accompanied by reflective debriefing exercises, proved to be an effective approach for bridging the gap between theoretical knowledge and its application in clinical practice. Therefore, the study prompts curriculum revisions to incorporate family assessment into nursing practices, as well as evidence-based strategies, such as learning activities that use SP or HFS technology to address and possibly reduce the theory-practice gap for graduates when entering clinical practice.

The use of family genograms and ecomaps during simulations showed statistical significance in the clinical practice environment. Additionally, there was a notable difference in student perceptions of simulation's effectiveness as a learning tool, and the Gelderen family care rubric proved valuable for evaluating student performance and providing constructive feedback. Overall, the research underscores the importance of family involvement in patient care and the benefits of simulation-based training for nursing students to improve their clinical skills. This research study adds to the literature on simulation-based nursing education. It provides empirical evidence of the potential to positively impact nursing practice and reduce the theory-practice gap for graduates entering clinical practice.

## Appendices

Appendix A

Part I: Personal/academic data

1. Age:

2. Academic level:

3. Marital status:

4. If you are married, do you have children?

• Yes ( ) • No ( )

If yes,

5. Number of children:

6. Do you have any experience with family assessment in client care?

• Yes ( ) • No ( )

If yes, what are the sources of information?

• Lecture ( ) • Workshop ( ) • Special reading ( ) • Others ( )

7. Do you have any working experience?

• Yes ( ) • No ( )

If yes, where:

Do you currently use family assessment in dealing with clients?

• Yes ( ) • No ( )

Appendix B

Van Gelderen simulation rubric: Communication, assessment and integration of family-based care (Stacey Van Gelderen, 2010)

• Positive characteristics: 3 points

• Characteristics needing improvement: 2 points

• Undesirable characteristics: 1 point

Evaluator notes

Nurse communication style (Rosenzweig et al., 2008)

• Communication was therapeutic and open-ended; attentive listening skills were used

• Communication was open-ended; distracted in listening skills; communication was perceived as rushed

• Communication was directive (one-way); advice-giving type of communication; listening was not used

Use of terminology

• Discussion and terminology were appropriate for the client/family

• Communication occasionally used medical jargon or the use of inappropriate terminology

• Communication used medical jargon and inappropriate terminology

Nurse positioning

• Nurse position was appropriate; positioned at eye level during interviews/conversations; felt respectful toward client/family

• The nurse position was appropriate at times; sometimes perceived as un-engaged

• The position was domineering and perceived as overpowering toward the client/family

Nurse eye contact

• Appropriate eye contact - Equal eye level - Respectful - Non-invasive - Attentive

• Did not maintain appropriate eye contact; was distracted with technical tasks

• Poor eye contact; directed away from family members

Family history and data collection method (Wright and Leahey, 2005)

• The nurse used a family genogram and ecomap to help identify family support and resources

• The nurse initiated a family genogram and ecomap but left it unfinished or the family felt rushed

• The nurse did not initiate a family genogram or ecomap to identify family support and resources

Addressing family issues and concerns

• Clarified understanding of client/family issues and concerns - Stressors - Needs - Resources - Support

• Inconsistent with clarification or did not address all clients/family issues and concerns - Stressors - Needs - Resources - Support

• Did not clarify or inquire about client/family issues and concerns

Addressing nursing involvement

• Clarified understanding from the client/family of their perceived needs/desires of nursing involvement in decision-making processes

• Identified options for nursing involvement, but did not clarify client/family needs/desires of involvement

• Did not clarify client/family perceived needs/desires for nursing involvement with decision-making processes

Addressing needs for follow-up care

• Discussed needs for follow-up care; informed and gave possible resources

• Discussed follow-up care, but was ambiguous about information and did not tailor it to the family’s needs

• Did not discuss the need for follow-up care

Offer of support and hope (Herth, 1991)

• Made a positive impression on the family by offering support and hope

• Made an indifferent/ambiguous impression toward the family

• The family is unsure of the nurse’s intent

• Family may have mixed emotions about perceived support and hope

• Made a negative impression on family; did not offer support or hope

Provided care based upon the "Family as Client’ approach (Hansen, 2005)

• Nursing care focuses on the assessment of all family members; family is in the foreground, and the client is considered in the background; family is seen as the sum of individual family members and the focus concentrates on each individual; family members are validated.

• Nursing care focuses on the assessment of the client. Family members are asked questions, but not assessed or included as part of care and assessment.

• Nursing care focuses on individual clients. Family is not included as part of the assessment. The individual is in the foreground and the family is in the background or not acknowledged at all. The focus of care is on the client alone. The family members are not validated.

Family health routines are assessed (Denham, 2003)

• The nurse investigates the family’s: - Routines - Behaviors - Values - Relationships - How crises and information affect the family - Celebrations - Traditions - Spirituality

Then, base nursing care on the family’s routines and strengths

• Nurse inquires about family health routines, but does nothing to embrace their individuality as part of their nursing care

• The nurse does not inquire about family health routines and does not base nursing care on the individual needs of the family

Total points: Possible: 33

Total Score: /33

Other general comments: ……………………………………………………………………………………………………………………………………………………………………………………………………………………………………………………………………………………………………………………………………………………………………………………………………………………

Appendix C

Nursing students' perceptions of the importance of family as client care

Survey questions

1. I have been a patient in a healthcare setting:

• Yes ( ) • No (Skip question 2) ( )

2. If yes, I felt my family members were respected and included in my care.

1. Strongly agree ( ) 2. Agree ( ) 3. Disagree ( ) 4. Strongly disagree ( )

3. I have been a family member of a patient within a healthcare setting.

• Yes ( ) • No (Skip question 4) ( )

4. How comfortable are you in working with families in a healthcare setting?

1. Very comfortable ( )

2. Comfortable ( )

3. Uncomfortable ( )

4. Very uncomfortable ( )

Please rate questions 5-13 using a scale of 1-4: (it will be used pre and post)

• Not Important

• Less Important

• Important

• Very Important

5. How important is it to include family members as part of the care of the patient?

6. How important is it to understand the family’s beliefs about health care?

7. How important is it for the nurse to interact with families in a healthcare setting?

8. How important is it for the nurse to collect family history during a patient admission?

9. How important is it for the nurse to address family issues and concerns during a patient

admission?

10. How important is it for the nurse to address the need for follow-up care during an admission assessment?

11. How important is it to offer support and hope to the family?

12. How important is it for the nurse to address family health routines?

13. How important is it for the nurse to address ethical and social justice inequities within family units?

Post-role-play simulation questionnaire

One week ago, you observed two simulated role plays of a nurse conducting an admission on a patient with a family member present. The following questions will refer to that simulated learning experience:

Students will be asked to rate their responses on the following:

Questions

• Not Important

• Less Important

• Important

• Very Important

1. How important is it to include family members as part of the care of the patient?

2. How important is it to understand the family’s beliefs about health care?

3. How important is it for the nurse to interact with families in a healthcare setting?

4. How important is it for the nurse to collect family history during a patient admission?

5. How important is it for the nurse to address family issues and concerns during a patient

admission?

6. How important is it for the nurse to address the need for follow-up care during an admission

assessment?

7. How important is it to offer support and hope to the family?

8. How important is it for the nurse to address family health routines?

9. How important is it for the nurse to address ethical and social justice inequities within family units?

Please rate questions 10-20 using a scale of 1-4:

No.

Statements

• Strongly disagree (1)

• Disagree (2)

• Agree (3)

• Strongly agree (4)

10. I felt the two nurse-family simulation role plays contributed toward my understanding of family as client care.

11. The simulation debriefing time (time spent talking about the scenarios) was beneficial to my learning.

12. Having the opportunity to practice family-focused care assessments in the nursing lab was important to me.

13. Having the opportunity to play the role of a family member during the practice time was an important piece of my learning about family members’ feelings.

14. I understand the use of family genograms in the clinical practice environment.

15. I feel the use of family genograms in the clinical practice environment is important.

16. I understand the use of family ecomaps in the clinical practice environment.

17. I feel the use of family ecomaps in the clinical practice environment is important.

18. Learning more about family as client care is important to me.

19. The role-plays enhanced my knowledge of ethical and social justice inequities within family units.

20. I would recommend this simulated family assessment experience for future nursing students.
